# Editorial: Trends in Digital Medicine

**DOI:** 10.3389/fmed.2020.00116

**Published:** 2020-04-03

**Authors:** Enrico Capobianco, Licia Iacoviello, Giovanni de Gaetano, Maria Benedetta Donati

**Affiliations:** ^1^Institute Data Science and Computing (IDSC), University of Miami, Miami, FL, United States; ^2^Department of Epidemiology and Prevention, IRCCS NEUROMED, Pozzilli, Italy; ^3^Department of Medicine and Surgery, University of Insubria, Varese, Italy

**Keywords:** digitalization, big data, electronic health records, precision medicine, disease management

*Trends in Digital Medicine* provided a series of contributed papers covering a variety of aspects currently defining the digital paradigm (1). Among the factors that were considered central to this Research Topic, a few were: (a) role Electronic Health Records (EHR) in public health and epidemiological studies, (b) applications of high-throughput genomics, (c) integration of omics at the experimental level, (d) impact of technologies ensuring accuracy according to the precision medicine paradigm.

In parallel, multi-source evidenced data represent the common instrument available to researchers for collecting, storing and analyzing impressive amounts of molecular and clinical data that together are determining transformative effects. In particular, the effect of revising the scientific method to deal with new types of complexity (data structures, computational efficiency, algorithmic optimality etc.) and the effect of advancing translational medicine toward the identification of new principles useful to define healthy vs. diseased individuals and predict their profiles and health/disease trajectories.

We asked to refer to a few basic questions, following widely debated topics (2–5) such as: (1). Has digitalization achieved a status of a disruptive transformation in medicine? (2). Do EHR show clear utility in the clinics providing superior support to patients? (3). Big Data and doctors, are their synergies of any help to face Big Killers?

The first question about digitalization was addressed by a few contributed papers, such as Assale et al. who examined the relevance of the notes produced in clinical practice whose unstructured nature prevents a profitable secondary use but whose potential is increasingly relevant in digitized facilities for improving data quality and thus enabling predictive inference models to be designed and implemented. Other examples were provided by Cazzaniga et al., who discussed cancer registries as main resources for real world data research, and by Geldof et al. who discussed sector-specific challenges in exploratory and hypothesis-driven studies aimed at assessing treatment effectiveness.

The second question on EHR was addressed by Riba et al., who reviewed current issues related to data integration and governance in the medical field, by Silverio et al., who discussed of CVD and the importance of harmonizing large data sets to build algorithms for targeting and personalizing treatments, and then by Pala et al., who examined in urban contexts the role of both environmental and socioeconomic factors influencing common pathological outcomes such as asthma. The study has introduced spatial methodologies cross-linking data from hospitalized patients and pollution plus other factors and then enabling stratifications based on conditions such as poverty and health insurance together with well-studied race and obesity.

The third question on the relationships between Big Data and Big Killers has been elaborated by three studies. Gialluisi et al. have discussed of healthy aging and in particular of biological age, a sort of latent feature of every organism that can be derived from the combination of various biomarkers (especially blood-based). Of relevance to screening and risk profiling practices, the concept has been applied to the Moli-sani, a prospective population-based cohort study of 24,325 subjects (35–99 years). In such context, the analysis of brain age via neuroimaging combined with epidemiological, environmental and genetic variables may be very useful to advancing knowledge on common age-related diseases such as Alzheimer.

Finally, Franchini et al. introduced a model called Individual Profile of Pathology (IPP) designed to stratify populations affected by chronic diseases. When such conditions are considered dynamically driven by evolving multifactorial processes, then IPP offers advantages in assessing surveillance programs and guiding public health policy makers.

## Author Contributions

EC wrote the article. All authors shared and approved the contents.

### Conflict of Interest

The authors declare that the research was conducted in the absence of any commercial or financial relationships that could be construed as a potential conflict of interest.
